# A novel de novo nonsense mutation in *SALL4* causing duane radial ray syndrome: a case report and expanding the phenotypic spectrum

**DOI:** 10.1186/s12920-023-01467-1

**Published:** 2023-02-24

**Authors:** Mobarakeh Ajam-Hosseini, Farshid Parvini, Abdolhamid Angaji

**Affiliations:** 1 Department of Cellular and Molecular Biology, Faculty of Biological Sciences, Kharazmi University, Karaj, Iran; 2 Department of Biology, Faculty of Basic Sciences, Semnan University, Semnan, Iran

**Keywords:** *SALL4* gene, Duane-raidal ray, Case report, Nonsense mutation, Iran

## Abstract

**Background:**

*SALL4*, a member of the *SALL* genes family, encodes a zinc-finger transcriptional factor that either activates or represses gene transcription depending on cell type during embryonic development. *SALL4* mutations cause extremely variable conditions including Duane-radial ray (DRR), Okihiro, Holt-oram, Acro-renal ocular and IVIC syndromes, all with autosomal dominant inheritance pattern. However, all these syndromes with different terminologies are actually the same entity termed *SALL4* related disorders.

**Case presentation:**

Herein, we examine an Iranian patient suspected to DRR syndrome which has not been previously described in the population. Whole-exome sequencing (WES) was performed to examine pathogenic genes in the proband. Subsequently, Sanger sequencing was used to confirm the mutation found. To elucidate the effects of the identified mutation, clinical data of patient was collected. Morever, the possible impact of the mutation found on the corresponding protein was evaluated using bioinformatics tools. WES identifed a novel de novo heterozygous nonsense mutation in exon 2 of *SALL4* gene (c.712 C > T:p.Q238X). Subsequently, segregation and phenotype-genotype correlation analysis as well as *in-silico* approaches confirmed the autosomal dominance inheritance and disease-causing nature of the identified mutation. In addition, studied patient had features not described previously, including kyphoscoliosis, dimple presacral sinus, barrel chest and artric disc (C6–C7). These manifestations could be additional characteristics of the growing phenotypic spectrum of *SALL4* related disorders.

**Conclusion:**

Our findings could extend the pathogenic mutations and phenotypic spectrum of *SALL4* related disorders. Such reports can also aid to conduct genetic counseling, prenatal diagnosis and clinical management for individuals at high risk of *SALL4* related disorders.

**Supplementary Information:**

The online version contains supplementary material available at 10.1186/s12920-023-01467-1.

## Background

The *SALL* genes, encoding zinc-finger transcription factors, are homologous to the Drosophila spalt gene and also present in mice. Four of which have been identified in humans, so far [1, 2]. The researchers have found that *SALL4* gene expression is higher in the embryonic stage, while its expression decreases in adulthood and is limited to the testis and ovaries [3]. On the other hand, Yong et al. [4] through loss of function studies on *SALL4* realized the vital role of this gene in cell survival and tumorigenesis. The *SALL4* gene, with four exons (3159 bp) and 18.14 kb length, is located on 20q13.13–q13.2 region, and encodes a homonymous transcription factor SALL4 with 1053 amino acids [5, 6]. This protein contains eight zinc finger motifs that three highly conserved C2H2 double zinc finger domains of the protein are evenly distributed, a single C2H2 motif is attached to the second domain and at N-terminal region contains an isolated C2H2 motif [7].

*SALL4*-related disorders show autosomal dominant inheritance pattern, so that haploinsufficiency of *SALL4* gene leads to Duane-radial ray (DRR), IVIC, Acro-renal ocular (ARO), and rarely, Holt-oram syndromes with approximately 40–50% of cases being caused by a de novo mutations [8, 9]. ARO syndrome is characterized by eye defects mainly as colobomas, radial ray malformations, and renal abnormalities [10]. DRR syndrome, also known as Okihiro syndrome, has highly variable clinical manifestations and shows incomplete penetration [11]. Additionally, it is worth mentioning that 70% of radial ray defects are associated with other abnormalities (syndromic) and 30% are isolated [12]. Duane malformation and radial ray disorders such as the limbs (especially thumb), hearing loss and anomalies of renal and anorectal, identified in this syndrome [5]. In patients with *SALL4* mutation, radial ray abnormalities account for 91.3%. In addition, duane retraction syndrome (DRS) is a congenital anomaly that leads to impaired eye movement due to abnormal growth of the cranial nerve VI [13]. About 1–5% of all cases of strabismus involve duane retraction syndrome [11]. IVIC syndrome was first identified in 1980 through allelic heterogeneity in the *SALL4* gene of six generations of a Venezuelan family with autosomal dominant pleiotropic traits. In general, the clinical manifestations reported in patients with IVIC syndrome are similar to those of DRRS affaceted patients, but phenotypes such as mild thrombocytopenia, leukocytosis (before age 50), and sensori-neural or conduction deafness, or both have not been observed in Okihiro syndrome. Therefore, it is difficult to distinguish between these two syndromes [10, 14]. However, all these syndromes with various names and terminologies are actually the same entity called *SALL4* related disorders.

In this study, we describe a novel de novo heterozygous nonsense mutation in *SALL4* gene segregating with duane radial ray syndrome in an Iranian family. Furthermore, the present study further expands the clinical symptoms associated with DRRS by describing the novel abnormal phenotypes manifested in the affected individual. A review of the literature is also provided. Beyond any doubt, such studies show that rapid progress in the field of high throughout sequencing allows more accurate and less expensive diagnosis of rare inherited disorders [15–17].

## Case presentation

**Fig. 1:**
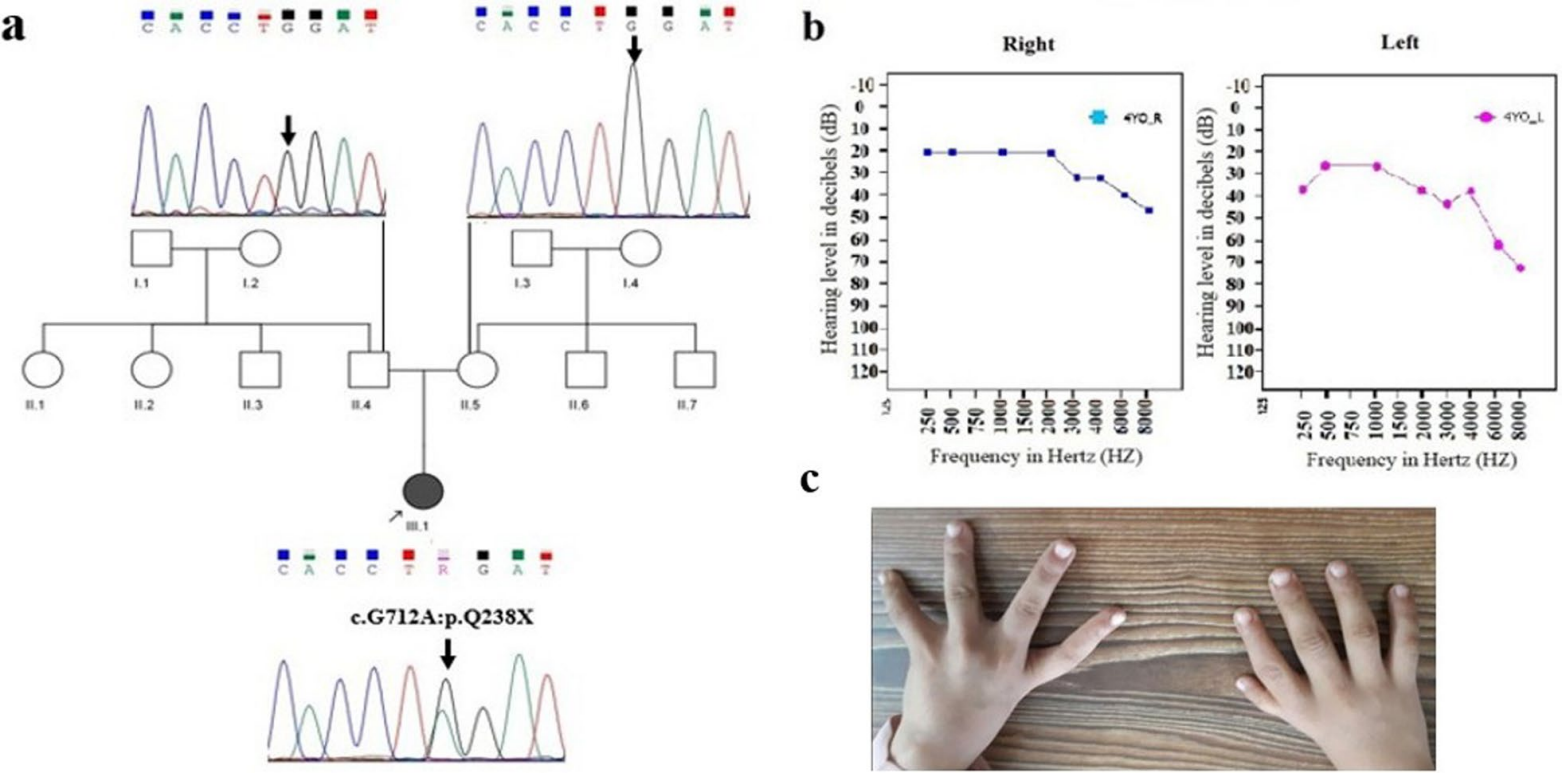
Family pedigree and results of audiologic evaluation of the proband (III-1). **a** Pedigree and segregation analysis of c.712 C > T: p.Q238X mutation in the *SALL4* gene. Sanger sequencing displayed that patient (III-1) is heterozygous and her healthy parents (II-4 and II-5) are normal for the identified mutation. **b** Audiograms of the affected proband which were obtained using pure tone audiometry with air conduction from frequencies 250 to 8000 Hz. **c** Patient's dysmorphic phenotypes. Left thumb aplasia, hypoplasia of right thumb, and mild syndactyly are evident

At the present study we explored the molecular mechanism of pathogenesis in a patient suspected to duane radial ray syndrome. The nonconsanguineous pedigree with an affected girl was recruited (Fig. 1a). The studied family is of Iranian origin located in Semnan province (central Iran). As shown in Fig. 1, proband was a 4-year-old girl with clinical symptoms of DRRS who results from a non-consanguineous marriage. Clinical and physical examinations of the patient showed craniofacial deformity, mild microcephaly, cleft palate, thumb aplasia of left hand, thumb hopoplasia of right hand (Fig. 1c), mild syndactyly in right hand, bilateral club foot and short leg, kyphoscoliosis, barrel chest, deep presacral sinus, anorectal abnormality, anal stenosis, constipation, and fecal incontinence.

Echocardiography diagnosed the patient with atrial septal defect. Additionally, the patient underwent audiometric and ophthalmic evaluation. This assessment showed moderate and severe hearing impairment in the right and left ear (Fig. 1b), respectively, as well as type III duan anomaly as deep-set eyes with partial strabismus of right eye. Cleft palate was surgically repaired. She did not show any impairments in the ultrasound evaluation of the kidney, liver, gallbladder and bladder. Transfontanelle ultrasound showed no evidence of hydrocephalus, as well. In addition, Fig. 1c shows the phenotypes of craniofacial deformity, mild microcephaly, mild syndactyly and thumb abnormalities in patient III-1. On the other hands, the patient underwent MRI of the brain, which showed normal structure in the supra, infratentorial, and midline areas. The cortical and white matter signals of the brain, pituitary gland, parasellar regions, base of skull, orbits and 7-8th nerve complexes were also unchanged. MRI of the cervical spine and thoracolumbar spine showed a partial atretic disc at the C7-C6 leve and the presence of lipoma at the end of the filum, however, other regions were normal. According to the experiment performed at 4 year of age of patient, the concentrations of IGF1, TSH and T4 were in the normal range (101 ng/ml, 3.4 µIU/ml and 10.5 µg/dl respectively), but the T3 resin uptake was slightly higher than the normal range (37.4%).

After obtaining informed consent, karyotype test was performed for the patient. Since no chromosomal abnormalities were detected, genomic DNA was extracted from blood sample of patient and her healthy parents using QIAamp DNA Blood Mini Kit (Germany) according to the manufacturer's instructions. To investigate the genetic cause of the wide range of abnormalities reported in the affected patient, whole-exome sequencing was used to enrich all exons of the protein-coding genes and a few other important genomic regions. The WES was performed for about 100 million reads, using the Illumina Hiseq2000 sequencer platform and Agilent SureSelect Human All Exon V7 kit (Agilent, Santa Clara, USA). The output raw data were converted from bcl to fastq files by Bcl2Fastq software (Bcl2Fastq 2.18.0.12; Illumina, Inc.). Then, Illumina sequencing adapters and all lowquality reads (< 80 bp) were filtered using fastp software (https://github.com/OpenGene/fastp). The clean reads (fastqc files) were mapped to the UCSC hg19 human reference genome using BWA software (v0.7.12r1044; http://biobwa.sourceforge.net/). Subsequently, the duplicated reads were removed using Picard software (v2.2.3; http://broadinstitute.github.io/picard/) and mapped reads were used for the detection of variants/mutations. For this purpose, data filtering was first performed based on frequency and then according to intronic, upstream, downstream, 3ʹ-UTR, 5ʹ-UTR, intergenic and other non-coding variants. At final step, synonymous mutations were also fltered. In general, test platform examined more than 95% of the targeted regions with sensitivity of > 99%. The results of WES were analyzed by bioinformatics tools BWA aligner [18], GATK [19], and Annovar [20] and public databases ClinVar, gnomAD, Kaviar, and GME. In addition, ACMG (American College of Medical Genetics) guidelines and local population database (BayanGene) with more than 4000 unrelated individuals were utilized. As control, 250 healthy individuals with the same ethnicity as the studied patient were also screened for the mutation found. Online bioinformatics tools MutationTaster, SIFT, and CADD_phred software were used to predict the likely pathogenic effects of the mutation. To investigate the effect of identified mutation on the SALL4 protein in terms of possible structural and functional changes compared to wild-type protein, SMART tool was used. In order to confirm the new mutation found, PCR and Sanger sequencing was performed. Primers was designed using the Primer3 program (https://primer3.ut.ee) as: F-5ʹ GGCTTCCAGCTTTCTGGCTG 3ʹ and R-5ʹ ACCAAGGTGGCGGTGAATCAG 3ʹ (PCR product: 280 bp). Subsequently, Chromas software was applied to analyze the results of Sanger sequencing.

**Fig. 2:**
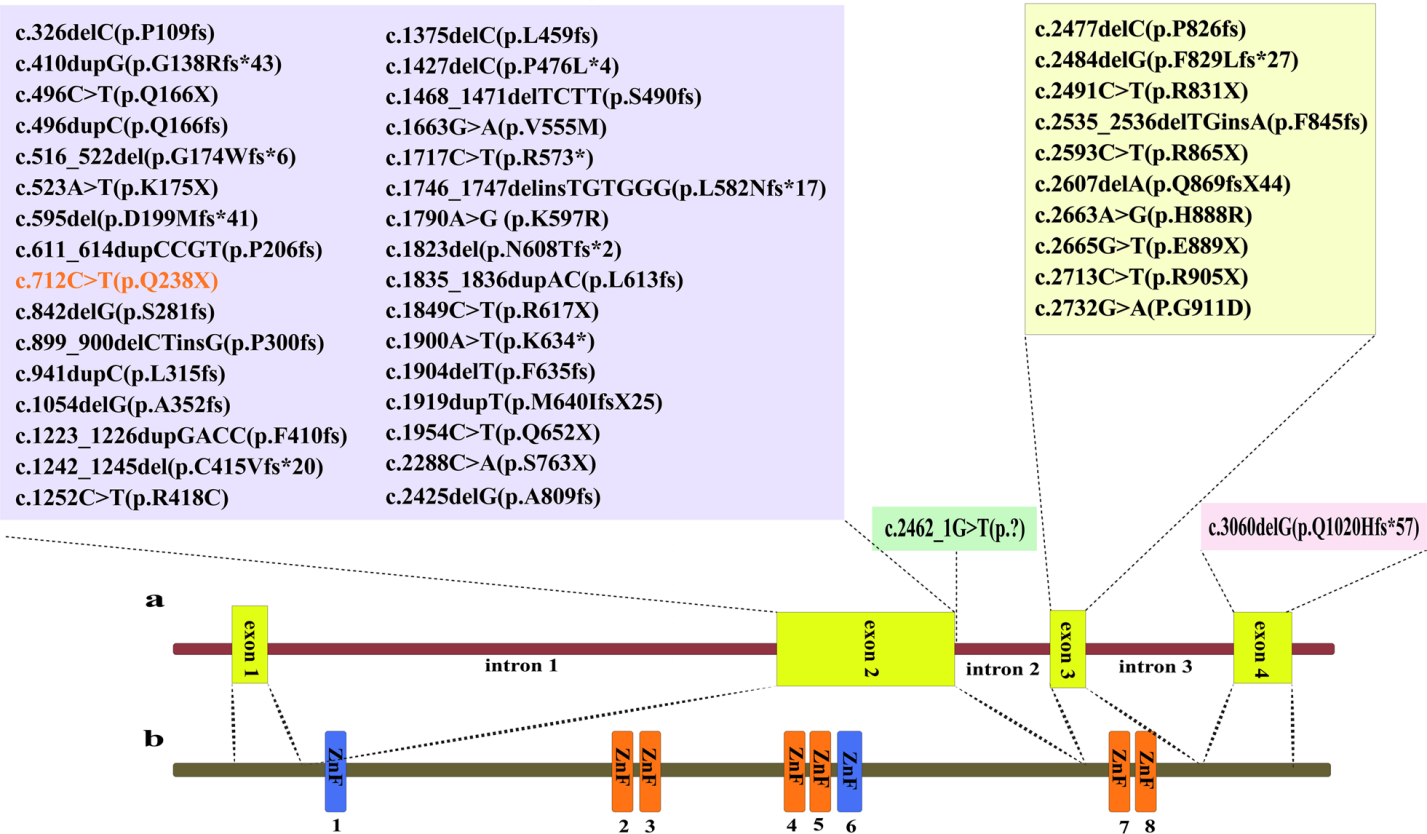
Schematic representation of the *SALL4*gene/protein structure. **a** Previous *SALL4* mutations were reported and the new mutation found (c.712 C > T;p.Q238X) is shown with a different color. **b** SALL4 consists of three C2H2 double zinc finger domains (orang) and two seperated C2H2 motifs (blue)

According to WES technique results, we identified a novel heterozygous stop-gain mutation in exon 2 of *SALL4* (NM_001318031); chr20:51791771: c.712 C > T: p.Q238X in the proband (patient III-1). The review of public databases and the local population database (bayangen) did not identify any previous reports of the identified mutation in the *SALL4* gene. In addition, none of the 250 healthy individuals (controls) with the same ethnicity as the studied patient showed the identified mutation, emphasizing the novelity of the mutation found. The identified mutation was predicted to be disease-causing by *in silico* approaches (Table 1). Furthermore, genotype-phenotype correlation analysis matched the observed phenotypes in patient with the detected novel mutation in the *SALL4* gene. As shown in Fig. 1a, the Sanger sequencing confirmed the existence of the mutation in the proband, and its absent in her healthy parents showing de novo origin of the mutation found. After audiological evaluation, moderate conductive hearing impairment was reported in the right ear and severe mixed hearing impairment in the left ear of the proband. In this assessment, the pure tone of the patient was measured at 250–8000 Hz (Fig. 1b). These data suggest that this new mutation could be the cause of DRR and IVIC syndromes with a wide range of abnormalities in the studied patient. As depicted in Fig. 2, the SALL4 protein has eight zinc finger motifs: three C2H2 double zinc finger domains which are highly conserved, the second of which has a single C2H2 zinc finger attached at its C-terminal end, and also an N-terminal C2HC zinc finger motif [6].

**Table 1:** Prediction of pathogenicity of the identified heterozygous mutation c.712 C > T:p.Q238X in the *SALL4* gene

Gene	Mutation	SIFT	Mutation taster	LRT-pred	CAAD-Phred (RefSeq)
*SALL4* (NM_001318031)	c.712C>T (Q238X)	D	D	N	36

*D* Damaging;* N* Neutral

## Discussion and conclusions

**Fig. 3:**
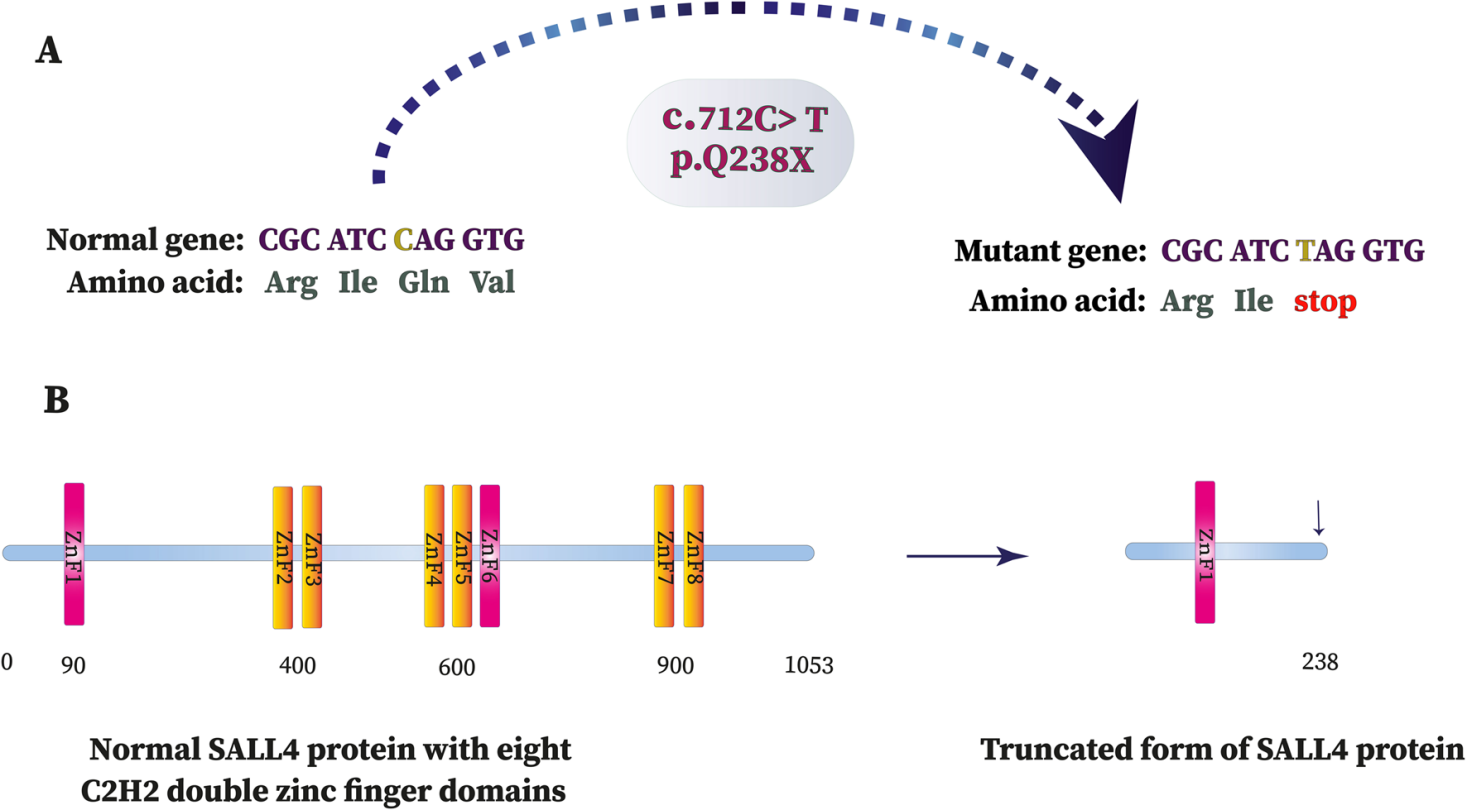
Schematic illustration of gene and protein changes due to nonsense mutation c:712 C > T:p.Q238X in *SALL4*gene. **a** The normal sequence of the gene and the corresponding amino acid sequence have displayed on the left. The nucleotide conversion of C to T is shown in different colors (yellow). The altered sequence and the corresponding truncated protein are depicted on the right. **b** 7 out of 8 C2H2 zinc finger domains of SALL4 protein are deleted due to novel de novo nonsense mutation c:712 C > T (p.Q238X)

In this study, we identified a new *SALL4* mutation (NM_001318031: exon 2: c.712 C > T: p.Q238X) in an Iranian affected patient suspected to DRRS. As far as we know, this is the first genetic study with respect to the *SALL4* gene mutations in Iranian populations. The identified mutation leads to the substitution of glutamine 238 with a stop codon (Q238X) in the SALL4 protein. Hence, this substitution alters the amino acid sequence and leads to a premature stop codon at position 238 with the complete loss of 819 out of 1053 amino acids in the wild type protein sequence. The deleted sequence contains 7 out of 8 C2H2 zinc finger domains including three highly conserved C2H2 double zinc finger domains and a single C2H2 zinc finger domain (Fig. 3) resulting to disruption of whole protein structure and function. In fact, this mutation is predicted to be responsible for the disease pathogenesis by either a truncated SALL4 protein affecting 7 functional C2H2 zinc finger domains or haploinsufficiency due to nonsense-mediated mRNA decay.

As previously pointed out, the *SALL4* gene belongs to a group of evolutionarily conserved genes called the spalt transcription factor family, which plays a critical role in regulating embryonic growth in many organisms. In addition, SALL4 transcription factor is known as a homeotic factor which is essential for the early growth of the posterior head and anterior tail regions in Drosophila [21]. As mentioned earlier, in human, mutations in the *SALL4* gene can cause DRR, IVIC, ARO, and HO syndromes that are associated with various malformations of several organ systems [10]. Based on observed phenotypes in the studied patient which previously listed in the patient section, these characteristics are highly matched with DRRS. Although, renal abnormalities have also been usual in affected patients [1, 11, 22], but this feature was not present in the patient we studied. Interestingly, new phenotypic features including kyphoscoliosis, dimple presacral sinus, barrel chest and artric disc (C6–C7) were also observed in our patient. However, it seems that all *SALL4* gene related disorders are allelic diseases with highly overlap phenotypes. Therefore, from now on, it is better to use the term *SALL4*-related disorders instead of referring to a specific disorder caused by the *SALL4*-gene mutation. In the case of our patient, since her parents did not show the mutation found, the origin of the mutation was de novo.

So far, 43 pathogenic and likely pathogenic variants have been reported in patients from Venezuela [23], Brazil [22], Germany [24], China [6, 25], Italy [26], Chile [3] and Turkey [12]. Recently, a frameshift mutation in exon 4 has been reported that leading to increasing in the length of the SALL4 protein from 1053 to 1076 amino acids [25]. However, like more than 73% of the mutations found in the *SALL4*, which occur mainly in the exon 2 of the gene, the mutation found in this study (as the forty-fourth identified mutation) is also located in exon 2. It confirms that the exon 2 is hotspot of mutation throughout the *SALL4* gene. However, it is not yet clear why different mutations in this pleiotropic gene lead to extremely variable characteristics in different affected patients. Except the type of *SALL4* mutation, it seems that involvment of SALL4 transcription factor in epigentics phenomena by recruiting the nucleosome remodeling and histone deacetylase complex (NuRD) [27], its different interactions with distinct genes and proteins [2, 28] and thus the genetic background of the SALL4 dependent protein network/pathways in patients are at least part of the answer to this question. Therefore, considering the precise pathogenesis mechanism and upstream/downstream target genes of SALL4 is of great importance in future studies to elucidate the exact cause of highly variable clinical symptoms of *SALL4* related patients.

In conclusion, we have identified a novel pathogenic heterozygous *SALL4* mutation in an Iranian family. The patient showed wide range of *SALL4* related disorders. In addition, new reported manifestations including kyphoscoliosis, dimple presacral sinus, barrel chest and artric disc (C6–C7) were expanded the phenotypic range of *SALL4* mutations. However, future reports would be essential in order to confirm these novel manifestations of the mutation found. Regarding to critical functions of SALL4 transcription factor in a wide variety of biological processes, future studies on either genetics or epigenetics aspects of upstream and downstream rugulations of *SALL4* might shed light the exact cause of these extremely variable characteristics in affected patients. Altogether, the results of this research provide a better understanding of *SALL4* mutations on phenotypic outcome and strengthens the clinical importance of this gene in affected patients of *SALL4* related disorders.

## Supplementary Information

**Additional file 1.** All variants detected in the studied patient.

## Data Availability

The identified variant in this research is accessible on the ClinVar repository under accession number "SCV002550878" for the *SALL4* gene mutation. Also, in addition to the pathogenic mutation found in *SALL4* gene, all other filtered variants/mutations found are available in the Additional file 1.

## Abbreviations

*SALL4*: Spalt like transcription factor 4
DRRS: Duane-radial ray syndrome
ARO: Acro-renal ocular
HOS: Holt-oram syndrome
AROS: Acro-renal ocular syndrome
IVIC: Instituto Venezolano de Investigaciones Cientificas
WES: Whole-exome sequencing
DRS: Duane retraction syndrome
ACMG: American College of Medical Genetics
